# Factors Predicting 150 and 200 Microgram Adenosine Requirement during Four Increasing Doses of Intracoronary Adenosine Bolus Fractional Flow Reserve Assessment

**DOI:** 10.3390/diagnostics12092076

**Published:** 2022-08-27

**Authors:** Thamarath Chantadansuwan, Jayanton Patumanond, Thammanard Charernboon, Dilok Piyayotai

**Affiliations:** 1 Department of Cardiology, Central Chest Institute of Thailand, Nonthaburi 11000, Thailand; 2 Center for Clinical Epidemiology and Clinical Statistics, Faculty of Medicine, Chiang Mai University, Chiang Mai 50200, Thailand; 3 Department of Clinical Epidemiology, Faculty of Medicine, Thammasat University, Pathum Thani 12121, Thailand; 4 Department of Internal Medicine, Faculty of Medicine, Thammasat University, Pathum Thani 12121, Thailand

**Keywords:** fractional flow reserve, intracoronary adenosine, percutaneous coronary intervention

## Abstract

Direct intracoronary adenosine bolus is an excellent alternative to intravenous adenosine fractional flow reserve (FFR) measurement. This study, during four increasing adenosine boluses (50, 100, 150, and 200 mcg), aimed to explore clinical and angiographic predictors of coronary stenotic lesions for which the significant ischemic FFR (FFR ≤ 0.8) occurred at 150 and 200 mcg adenosine doses. Data from 1055 coronary lesions that underwent FFR measurement at the Central Chest Institute of Thailand from August 2011 to July 2021 were included. Baseline clinical and angiographic characteristics were analyzed. The FFR ≤ 0.8 occurred at adenosine 150 and 200 mcg boluses in 47 coronary lesions, while the FFR ≤ 0.8 occurred at adenosine 50 and 100 mcg boluses in 186 coronary lesions. After univariable and multivariable logistic regression analyses, four characteristics, including male sex, younger age, non-smoking status, and FFR procedure of RCA, were predictors of the occurrence of FFR ≤ 0.8 at adenosine 150 and 200 mcg doses. Combining all four predictors as a predictive model resulted in an AuROC of 0.72 (95% CI: 0.68–0.76), an 86% negative predictive value. Comparing these four predictors, the FFR procedure of RCA gave the most predictive power, with the AuROC of 0.60 (95% CI: 0.56–0.63).

## 1. Introduction

These days, fractional flow reserve (FFR) measurement via coronary catheter is the reference standard for physiological assessment of the significant myocardial ischemic status from any coronary stenotic lesions. FFR measurement can guide physicians on which stenotic lesions at which coronary vessels should undergo myocardial revascularization [1,2]. Given the popularity of the FFR procedure worldwide, the European Society of Cardiology (ESC) stated the recommendation in the guideline for myocardial revascularization 2010 that the FFR should be performed in multivessel PCI, especially in cases without evidence of myocardial ischemia from non-invasive imaging stress test (class of recommendation I) [3]. Afterward, later guidelines from the ESC and the American College of Cardiology (ACC) have been established as further indications for the FFR procedure [4,5,6]. 

Current standard practice regarding the route of adenosine administration during the FFR measurement is an infusion method via a central vein, which is inconvenient due to a large amount of adenosine and central venous access catheter requirement, either at the groin or neck region [5,6,7,8,9]. For this reason, direct intracoronary adenosine bolus at different doses has been an alternative and widely used method by physicians worldwide [10,11,12,13,14,15,16,17,18,19]. However, the proper doses of direct intracoronary adenosine FFR measurement were unspecified until the ACC had stated the recommendation in the expert analysis part in 2017 [20].

At our institute, the Central Chest Institute of Thailand (CCIT), physicians have been using direct intracoronary adenosine FFR measurement in coronary stenotic lesions with 30–90% diameter stenosis since August 2011. Almost half of the protocols used the four increasing adenosine bolus doses (50, 100, 150, and 200 mcg). Although preferring the four increasing adenosine bolus protocol, due to the situation of case loading or the need to reduce the procedural time, physicians sometimes performed only one or two doses of adenosine injection, which caused suspicion in the accuracy of the FFR result as the stage of maximal hyperemia might be unobtained. 

In this study, during the four increasing doses, the lower dose of adenosine was defined as 50 and 100 mcg, whereas the higher dose was 150 and 200 mcg boluses. While doing the test, each step of increasing adenosine dose will give a ratio of pressure distal to the stenotic lesion divided by central aortic pressure (Pd/Pa), which will be interpreted as clinically significant ischemia if the Pd/Pa ≤ 0.8. The lowest Pd/Pa from the increasing adenosine boluses would be the FFR of that coronary stenotic lesion. In our study, the results of FFR in coronary stenotic lesions may be divided into three categories: (1) the FFR > 0.8; (2) the FFR ≤ 0.8 occurring at higher adenosine bolus doses; (3) the FFR ≤ 0.8 occurring at lower adenosine bolus doses. This study aimed to explore clinical and angiographic predictors of coronary stenotic lesions requiring a higher dose of intracoronary adenosine bolus (150, 200 mcg) to demonstrate ischemic response. In other words, coronary stenotic lesions with Pd/Pa > 0.8 at the lower dose; however, it turned to ≤0.8 at the higher dose of adenosine bolus. 

## 2. Materials and Methods

### 2.1. Study Design and Setting

The present study is a retrospective, cross-sectional, single-centered study conducted at the Central Chest Institute of Thailand, or CCIT, a tertiary care hospital with 333 in-hospital beds specializing in cardiopulmonary disease. Written informed consent was obtained from each patient before performing coronary angiography, FFR, and PCI procedures.

### 2.2. Patient Population

From August 2011 to July 2021, in a retrospective cohort, 1176 patients with 1288 coronary lesions underwent angiographic and invasive physiologic assessment by FFR before the intervention and were included in the current analysis. The study population included chronic coronary syndrome (CCS), patients with a history of unstable angina (UA), and non-ST elevation myocardial infarction (NSTEMI) within three months. CCS was defined as angina chest pain on exertion with a stable pattern for at least three months preceding coronary angiogram. Unstable angina and NSTEMI were defined according to Braunwald classification [21] and the universal definition of MI [22]. All patients were between 34 and 85 years of age and had at least one target vessel with 30–90% of coronary angiographic diameter stenosis seen upon the physician’s visual estimation. This study analyzed coronary artery stenotic lesions in patients with the following criteria.

Inclusion criteria:

- Age more than 18 years old.
- Underwent FFR measurement in the left or right coronary artery with complete four increasing intracoronary adenosine bolus protocol (50, 100, 150, 200 mcg). In case of receiving less than four doses (incomplete protocol), the patient needed to receive at least one adenosine bolus of the lower dose (either 50 or 100 mcg) plus at least one adenosine bolus of the higher dose (either 150 or 200 mcg).

Exclusion criteria:

- Underwent FFR measurement with intravenous adenosine infusion protocol.
- Patients with aorto-ostial (within 3 mm) lesions of left main or right coronary artery, diffuse coronary lesions, or culprit lesions of unstable angina or acute NSTEMI.
- Patients within the first four days of acute ST-segment elevation myocardial infarction.

### 2.3. Fractional Flow Reserve (FFR) Measurement

After engaging the coronary ostium with a guiding catheter, at least two orthogonal views of controlled angiograms were done to demonstrate target stenotic lesion that required FFR measurement. A 0.014-inch pressure monitoring guidewire (PrimeWire-Prestige, Volcano Corporation, San Diego, CA, USA) was zeroed ex vivo. Routine nitroglycerine at the dose of 100 or 200 mcg was administered intracoronary to abolish epicardial vasoconstrictor tone [9,20]. Then, contrast medium was flushed from the guiding catheter with normal saline. This was followed by the pressure wire being inserted until the pressure sensor of the pressure wire reached one or two millimeters distal to the tip of the guiding catheter. After equalizing the pressure between the pressure sensor of the pressure wire and the tip of the guiding catheter, subsequently, the wire was advanced further into the target coronary artery until the pressure sensor was located two to three centimeters distal to the lesion segment to record the mean distal coronary artery pressure (Pd) [15]. Simultaneously, mean aortic pressure (Pa) was recorded from the tip of the guiding catheter. The introducing needle was outside the Y connector during equalization and measurement of FFR. While doing the test, the Pd/Pa ratio at each intracoronary adenosine bolus during the four increasing adenosine bolus protocols (50, 100, 150, 200 mcg) was recorded as Pd/Pa_50_, Pd/Pa_100_, Pd/Pa_150_, Pd/Pa_200_, respectively. The lowest Pd/Pa ratio achieved represented the Pd/Pa ratio at the maximal hyperemic state and accounted for the FFR of that coronary stenotic lesion. The FFR value and the adenosine bolus dose for which the FFR occurred were also recorded. If the lowest Pd/Pa ratios were repeatedly obtained during the bolus protocol, the smallest amount of adenosine bolus of which the lowest Pd/Pa firstly occurred would be taken into account. For example, if the Pd/Pa_50_, Pd/Pa_100_, Pd/Pa_150_, and Pd/Pa_200_ were 0.82, 0.78, 0.78, and 0.78, respectively, the lowest Pd/Pa, or the FFR of that stenotic lesion, would then be 0.78 and was recorded as having occurred at the 100 mcg adenosine dose. Significant FFR was defined as ≤0.80 [3,23]. Lesions receiving intracoronary adenosine 50, 100, 150, and 200 mcg boluses were referred to as having received the complete four increasing adenosine boluses. In contrast, lesions that received the incomplete four increasing adenosine boluses meant lesions received less than four adenosine boluses but received at least one dose of the lower dose (50, 100 mcg) and at least one dose of the higher dose (150, 200 mcg). All lesions that underwent the complete four escalating intracoronary bolus doses of adenosine were included in the primary and secondary post-estimation analyses. Apart from the complete four increasing doses protocol, other incomplete four doses protocols that met the inclusion criteria were also collected for the current analysis.

### 2.4. Visual Estimation (VE) of Coronary Stenotic Lesions

While performing coronary angiography, each vascular segment of the coronary artery was recorded in two orthogonal or nearly orthogonal views to avoid missing important diagnostic information about eccentric stenosis. The narrowest view of the stenotic lesion was considered. The operator then estimated the percent coronary diameter stenosis visually by comparing the diameter of the narrowing point with the diameter of the adjacent normal vascular segment. If the two diameters’ ratios were about 1/2 or 3/4, the percent diameter stenosis would be 50% and 75%, respectively [24]. Therefore, the narrowing that was almost 3/4 of the adjacent normal vascular segment would be called 70% diameter stenosis. Narrowing that was less than 1/2 of the adjacent normal vascular segment would be called 30–40% diameter stenosis and narrowing that was more than 3/4 of the adjacent normal vascular segment would be called 80–90% stenosis, respectively. In this study, significant percent diameter stenosis of any stenotic lesion was more than 50% in the left main coronary artery or more than 70% in non-left main coronary artery [4].

### 2.5. Study Size Estimation

From our pilot data survey from August 2011 to July 2013, the results of 228 coronary stenotic lesions that underwent FFR measurement revealed that the FFR (the lowest Pd/Pa ratio) occurred at the lower-dose adenosine bolus (50 or 100 mcg) in 111 lesions and occurred at the higher-dose adenosine bolus (150 or 200 mcg) in 117 lesions. Sample size requirement by comparing clinical and angiographic characteristics between the two mentioned groups, using two-sample comparison of means and two-sample comparison of proportions, is shown in the study’s Supplementary Table S1. Considered statistically significant if a two-sided alpha error (*p*-value) was <0.05 with an 80% statistical power, the feasible variables that did not require too many sample sizes and had the potential to answer the primary research question were age, BMI, DM, and FFR procedure of the left coronary artery versus FFR procedure of the right coronary artery. Initially, we decided to include at least 460 coronary lesions per group.

### 2.6. Data Collection and Predictors

All data and predictors in this study were reported following the STROBE guidelines [25]. Clinical predictors included age, sex, BMI, comorbid diseases (diabetes, hypertension, hyperlipidemia), smoking history, and the clinical presentation of ischemic heart diseases (chronic coronary syndrome, unstable angina, non-ST segment elevation myocardial infarction). Angiographic predictors included FFR of left coronary artery versus right coronary artery, part of coronary vessels (proximal, mid, distal), percent diameter stenosis by visual estimation, and triple vessels disease. For practically applying the results of this study, we divided the age predictor according to the WHO definition of the elderly into two groups (younger group if age < 65 and elderly group if age ≥ 65 years) [26]. We also divided the BMI predictor according to the CDC statement that defined overweight as being when BMI ≥ 25 kg/m^2^, and severity of percent diameter stenosis according to the ACCF/AHA/SCAI 2011 Guideline for percutaneous coronary intervention that defined significant coronary artery stenosis as requiring further revascularization when visual estimation percent stenosis was ≥70% in non-LM and ≥50% in LM stenosis [4]. 

### 2.7. Statistical Analysis

The categorical data were presented as frequency and percentage. The continuous variables were reported as a mean ± standard deviation. Differences in categorical data of each predictive factor between coronary stenotic lesions with the occurrence of FFR ≤ 0.8 at the higher adenosine dose (150, 200 mcg) and the occurrence of FFR ≤ 0.8 at the lower adenosine dose (50, 100 mcg) were examined using Fisher’s exact test. Whereas differences in continuous variables between the two mentioned groups were assessed using the unpaired Student’s *t*-test. Univariable analyses were used to examine the relationship between each predictive factor of the total 12 predictive factors and the occurrence of FFR ≤ 0.8 at the higher-dose intracoronary adenosine procedure of FFR measurement. Multivariable analyses by binary logistic regression analysis with cluster robust method were used to assess whether predictive variables were still statistically significant when adjusted for other variables significantly associated with the occurrence of FFR ≤ 0.8 at the higher dose in the univariable analyses. In addition, the selection of potential predictors for the final predictive model was based on the statistical significance of the univariable analysis. All predictors with a univariable *p*-value less than 0.20 were included in a multivariable logistic model [27]. Then, predictors with a *p*-value more than 0.05 and an odds ratio close to 1.0 were sequentially removed from the model in a backward fashion. The reason we used binary logistic regression analysis in this research due to the endpoint of interest was the occurrence of FFR ≤ 0.8 at the higher dose (occurred vs. not occurred). Considering the repeated measurement of the Pd/Pa value after escalating injection of intracoronary adenosine in the same coronary lesion, the variance correction by cluster robust was applied. All tests were two-tailed. A *p*-value of <0.05 was considered statistically significant. The strength of the relation between predictive factors and the endpoint of interest was presented by the odds ratio, while the predictive power of these factors was presented by the area under the receiver operating characteristic (AuROC) curve. Finally, sensitivity analysis by testing the final predictive model in a subgroup of coronary lesions that received only the complete four increasing adenosine boluses was performed. All analyses were done using STATA/SE 16 software package (Stata Corp LP, College Station, TX, USA). 

## 3. Results

**Figure 1:**
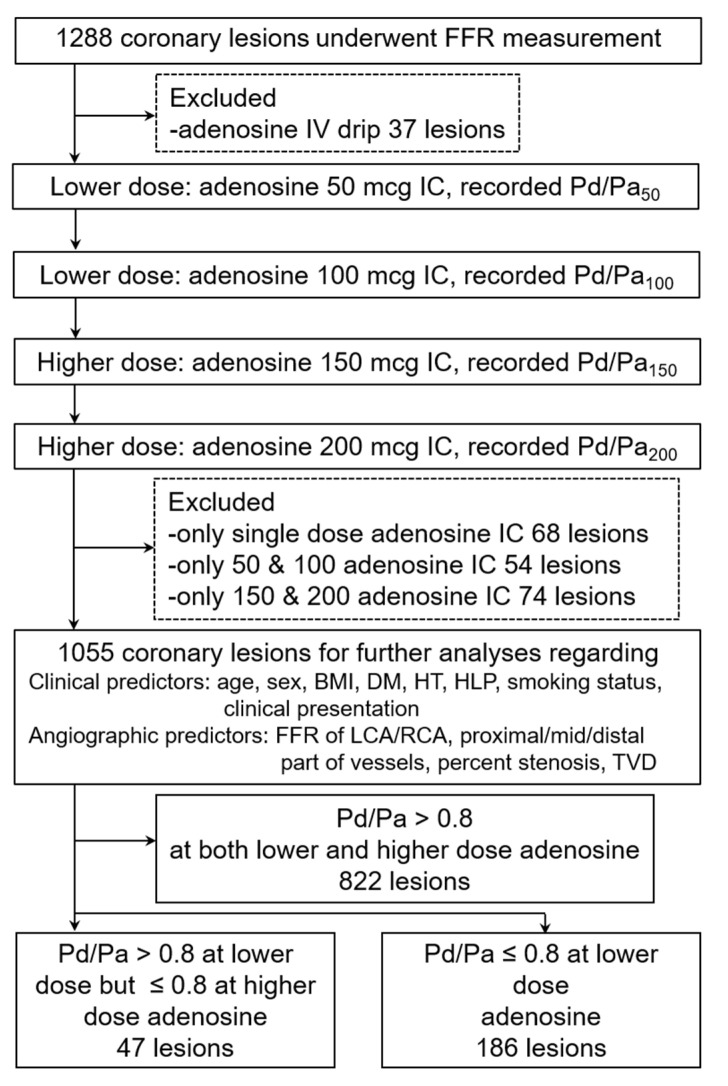
Study flow chart. FFR—fractional flow reserve; IV—intravenous; mcg—microgram; IC—intracoronary; Pd/Pa—ratio of pressure distal to coronary lesion divided by pressure of aorta; BMI—body mass index; DM—diabetes mellitus; HT—hypertension; HLP—hyperlipidemia; LCA—left coronary artery; RCA—right coronary artery; TVD—triple vessels coronary artery disease.

The study flow chart is demonstrated in Figure 1. 

From the study flow chart (Figure 1), total coronary stenotic lesions that met the inclusion criteria for further analyses numbered 1055 lesions, including lesions that received the complete four increasing adenosine boluses (391 lesions) and lesions that received the incomplete four increasing adenosine boluses (664 lesions) (328 coronary lesions received two bolus doses and 336 coronary lesions received three bolus doses).

**Figure 2:**
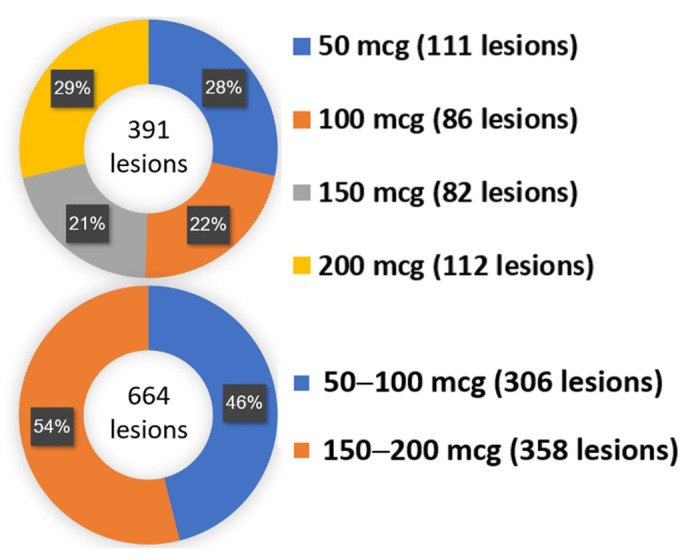
Donut chart summary: in which dose of intracoronary adenosine did the FFR occur? mcg—microgram.

From the upper donut chart in Figure 2, in coronary stenotic lesions that received the complete four adenosine boluses dose, the number of lesions that had obtained their lowest Pd/Pa (final FFR of that stenotic lesion) at 50, 100, 150, and 200 mcg adenosine bolus doses were 111, 86, 82, and 112 lesions, respectively. Meanwhile, for coronary stenotic lesions that received the incomplete four increasing adenosine dose, the lower donut chart in Figure 2 shows that the lowest Pd/Pa were obtained at the lower (50, 100 mcg), and the higher (150, 200 mcg) adenosine bolus doses for 306 and 358 lesions, respectively. 

This research involved FFR measurement of the left main and non-left main coronary stenotic lesions. The number of each coronary segment that underwent FFR measurement and percent diameter stenosis of the target lesion are shown in Table 1 and Table 2, respectively.

**Figure 3:**
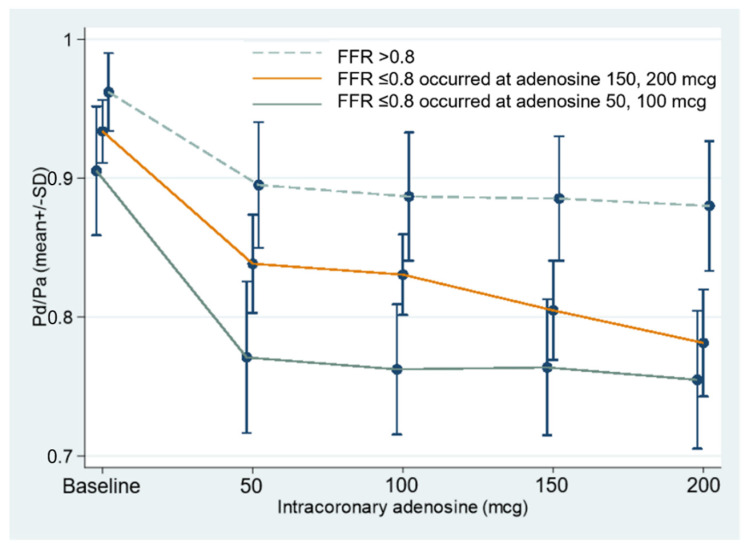
Three categories of changes in Pd/Pa value during FFR measurement: FFR > 0.8 (822 lesions), FFR ≤ 0.8 occurring at adenosine 150, 200 mcg (47 lesions), and FFR ≤ 0.8 occurring at adenosine 50, 100 mcg (186 lesions). FFR—fractional flow reserve; Pd/Pa—ratio of pressure distal to coronary lesion divided by pressure of aorta; mcg—microgram.

After measuring baseline Pd/Pa of each stenotic lesion, escalating intracoronary adenosine boluses as in the study flow chart were injected; Pd/Pa at each bolus dose were recorded and categorized into three characteristics of results as in Figure 3. This figure demonstrates the mean ± SD of baseline Pd/Pa, Pd/Pa_50_, Pd/Pa_100_, Pd/Pa_150_, and Pd/Pa_200_ of three divided groups with the FFR > 0.8 (822 lesions), the occurrence of FFR ≤ 0.8 at the higher dose (47 lesions), and the occurrence of FFR ≤ 0.8 at the lower dose (186 lesions).

Clinical and angiographic characteristics of coronary stenotic lesions with FFR ≤ 0.8 at the higher dose and FFR ≤ 0.8 at the lower dose were compared, as demonstrated in Table 3. The mean FFR of coronary stenotic lesions with FFR ≤ 0.8 at the higher dose was significantly higher than those with FFR ≤ 0.8 at the lower dose (0.78 ± 0.04 vs. 0.75 ± 0.05, *p* < 0.001), and coronary stenotic lesions with FFR ≤ 0.8 at the higher dose were found to be more common in younger age (<65 years old), non-hyperlipidemia, and patients who underwent FFR of RCA.

Table 4 demonstrates crude and adjusted odds ratio of predictors of coronary stenotic lesions with FFR ≤ 0.8 at the higher dose. Younger age, non-hyperlipidemia, and FFR procedure of RCA were significant predictors in univariable analysis. However, in multivariable analysis, the significant predictors turned into male sex, non-hyperlipidemia, non-smoking status, and FFR procedure of RCA.

After the results of multivariable analysis, we tried to select significant predictors to create a predictive model. Firstly, we excluded non-hyperlipidemia from our model. Considering data from Table 3, the incidence of non-hyperlipidemia in coronary stenotic lesions with FFR ≤ 0.8 at the higher dose was relatively low (3 from 47 coronary lesions). Moreover, the incidence of non-hyperlipidemia in coronary stenotic lesions with FFR ≤ 0.8 at the lower dose was even lower (only 1 from 186 coronary lesions). The difference in the proportion of non-hyperlipidemia between the two groups produced exaggerated crude and adjusted odds ratios compared with other predictors in Table 4. After that, secondly, backward elimination method resulted in the remaining final four predictors (male sex, younger age, non-smoking status, and FFR procedure of RCA). Table 5 demonstrates the adjusted odds ratio, *p*-value, and predictive power by AuROC of each predictor. Thirdly, we performed a sensitivity analysis in a subgroup of coronary stenotic lesions that received only the complete four increasing adenosine bolus, and the results were concordant with the main results (Supplementary Tables S2-1–S2-3).

**Figure 4:**
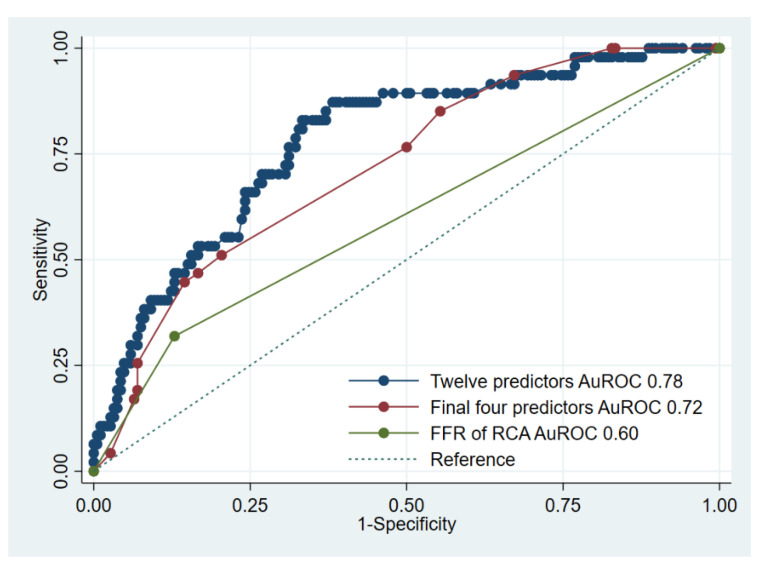
Comparison of area under receiver operating characteristic curve (AuROC) between all 12 predictors, final 4 predictors, and only single predictor (FFR procedure of RCA) in predicting the occurrence of FFR ≤ 0.8 at adenosine 150, 200 mcg. FFR—fractional flow reserve; RCA—right coronary artery.

Figure 4 illustrates the ROC curves of the entire model (AuROC 0.78, 95% CI: 0.74–0.81), the parsimonious model with the final four predictors (AuROC 0.72, 95% CI: 0.68–0.76), and the predictive power of the predictor FFR procedure of RCA (AuROC 0.60, 95% CI: 0.56–0.63). 

As our results showed that the FFR procedure of RCA gave the highest predictive power among our four statistically significant predictors, we explored the baseline Pd/Pa and Pd/Pa at each dose of intracoronary adenosine, comparing the FFR procedure of RCA (262 lesions) and LCA (793 lesions), and found that Pd/Pa_150_ and Pd/Pa_200_ of the FFR procedure of RCA still dropped down further. However, the FFR procedure of LCA did not have this appearance. Baseline Pd/Pa, Pd/Pa_50_, Pd/Pa_100_, Pd/Pa_150_, and Pd/Pa_200_ of the FFR procedure of RCA were 0.97 ± 0.03, 0.89 ± 0.06, 0.89 ± 0.06, 0.88 ± 0.06, and 0.87 ± 0.07, respectively, whereas baseline Pd/Pa, Pd/Pa_50_, Pd/Pa_100_, Pd/Pa_150_, and Pd/Pa_200_ of the FFR procedure of LCA were 0.94 ± 0.04, 0.86 ± 0.07, 0.85 ± 0.07, 0.85 ± 0.07, and 0.85 ± 0.07, respectively (Supplementary Figure S1). Furthermore, we also examined the cumulative frequency of ischemic FFR (FFR ≤ 0.8) that occurred at each dose of adenosine bolus, as reported in Supplementary Figure S2. Finally, we explored the number of coronary stenotic lesions in which the lowest Pd/Pa value was obtained during the lower (50, 100 mcg) and the higher (150, 200 mcg) adenosine bolus dose (Supplementary Figure S3a) and the number of coronary stenotic lesions in which the FFR ≤ 0.8 occurred at the higher (150, 200 mcg) adenosine bolus during the FFR of RCA vs. FFR of LM/LAD/LCX (Supplementary Figure S3b).

## 4. Discussion

The final predictors from our parsimonious model included male sex, younger age, non-smoking status, and FFR procedure of RCA. The odds ratio and AuROC with 95% CI of each predictor are presented in Table 5. We searched prior published articles to explain the plausible underlying mechanism of our results and found some, as in the following. First, the impact of gender on FFR measurement was questioned in the research of Kim SH. et al. [28] and Fineschi M. et al. [29]. The study of Kim HS. et al. [28], including the two-year data of 744 men and 261 women from the FAME study [30], which found that FFR values were significantly lower in men than in women, and the proportion of FFR ≤ 0.8 was higher in men than in women for lesions with 50–90% stenosis. In addition, the study of Fineschi M. et al. [29], including 317 intermediate coronary stenoses (40–70% stenosis by visual estimation), found that while resting, Pd/Pa was not different between gender; however, in response to adenosine 100 mcg for LCA and 60 mcg for RCA, a significantly larger ΔPd/Pa and a significantly lower FFR were observed in males. The possible explanation for this was differences in microvascular reactivity and myocardial mass between gender. In comparison, our results revealed, in the same direction, that the male gender was a predictor to predict Pd/Pa declining to ischemic Pd/Pa (Pd/Pa ≤ 0.8) after the higher-dose adenosine injection. Second, the effect of age on the FFR evaluation was in the study by Lim HS. et al. [31] and Mejia-Renteria H. et al. [32]. The study of Lim HS. et al. [31], comparing 512 patients enrolled in the FAME [30] study <65 years old to the 493 patients ≥65 years old, found that younger patients had lower FFR in vessels with 50% to 90% stenosis, and the proportion of FFR ≤ 0.8 in vessels with 71% to 90% stenosis was significantly higher in younger compared to elderly patients. Subsequently, in the study of Mejia-Renteria H. et al. [32], analyzing the intravascular physiology of 514 coronary stenotic lesions with 30% to 90% stenosis, revealed that FFR values increased progressively with patient age, potentially associated with age-related changes in the coronary microcirculation. Our study found that younger age was associated with Pd/Pa declining to ischemic Pd/Pa (Pd/Pa ≤ 0.8) after the higher dose adenosine injection. Third, the detrimental effect of smoking was mentioned in the study of Miyazaki T et al. [33]. This study, which included intermediate coronary stenotic lesions in 54 smokers and 43 nonsmokers, found that smoking was an independent predictor of coronary microvascular dysfunction. In comparison, our study revealed the predictive ability of Pd/Pa lowering to ≤0.8 after the higher dose of adenosine, which might be from a better response by microcirculatory dilatation after adenosine injection in non-smoking patients. Fourth, the most potent predictor in our observational study was the FFR procedure of RCA. We searched the previous literature regarding the impact of the difference in the left or right coronary artery on the FFR results and found at least two studies that explained hemodynamic differences in pressure waveforms between the LCA and RCA. The first one by Hadjiloizou N. et al. [34], including 20 patients who underwent measured simultaneous pressure and doppler flow velocity in the left main and proximal right coronary arteries, found that the flow velocity waveforms were different. The study stated that coronary flow velocity in the left main was predominantly in the diastolic phase and the diastolic flow velocity was lower in the RCA than in the left main. The second one by Wada K et al. [35], including 347 coronary vessels that underwent FFR measurement of the LCA and RCA, found the main findings as follows: (1) the pressure waveform distal to the stenotic lesions differed between the LCA and RCA; (2) in the LCA, diastolic pressure was predominantly decreased distal to the stenosis; (3) in the RCA, systolic pressure was predominantly decreased distal to the stenosis; (4) the different changes in distal coronary artery pressure waveforms between the LCA and RCA could be one of the causes of lower FFR values in the LCA, compared to the RCA. Whether the different changes in distal coronary artery pressure waveforms between the LCA and RCA also caused the likelihood of the RCA to decline in Pd/Pa value to ≤0.8 after the higher-dose adenosine injection as in our study was unknown. However, from our observational results, the FFR procedure of RCA was the most potent predictor in our parsimonious model.

The present research on factors predicting 150 and 200 mcg adenosine requirement during direct intracoronary adenosine boluses FFR measurement has found the following observations. Firstly, in overall FFR measurement, the lowest Pd/Pa value occurred in the lower or the higher dose adenosine bolus in the same proportion (Figure 2). This stems from the fact that coronary vasodilatory response to intracoronary adenosine administration is dose-dependent [36]. When using a greater amount of adenosine, coronary microcirculatory capillaries dilatation will be more pronounced, while the Pd/Pa value will be lower. One usually presumes that when using escalating intracoronary adenosine boluses from a lower to higher dose, Pd/Pa value should be lowest when using the maximum dose. However, from this research, we found that the lowest Pd/Pa value occurred either in the lower- or the higher-dose adenosine bolus in the same proportion (Figure 2). Secondly, we found evidence of positive ischemic results (FFR or the lowest Pd/Pa ≤ 0.8) in 233 from 1055 coronary lesions (22.1%). In coronary lesions with positive ischemic results, the FFR occurred at the lower adenosine injection in most lesions (186/233 lesions or about 4/5 of lesions). Furthermore, we found the Pd/Pa results that were >0.8 during lower dose adenosine injection but turned to ≤0.8 during higher-dose adenosine injection in minor lesions (47/233 or about 1/5 of lesions). Thirdly, administering adenosine at the higher dose resulted in an increasing the cumulative incidence of ischemic FFR (FFR ≤ 0.8) in both the left and right coronary arteries (Supplementary Figure S2). Furthermore, from our observation, the FFR procedure of RCA was more susceptible to increasing the dose of adenosine bolus than LCA in both the further dropping down of Pd/Pa (Supplementary Figure S1) and increasing the cumulative frequency of ischemic FFR (Supplementary Figure S2). Fourthly, when considering each predictor from our final predictive model, the most potent predictive factor was the FFR procedure of RCA. We found that the FFR procedure of RCA was one of the potential predictors that caused the FFR obtained at the higher dose of intracoronary adenosine during our sample size estimation period (Supplementary Table S1) when we started the present research. Interestingly, after finishing the research, this predictor was still the most potent predictive factor in our final predictive model (Table 5). We hoped the consistency of this predictor from our pilot survey to the final results made this predictor a reliable predictor. Fifthly, the further dropping down ability of Pd/Pa when using higher dose 150 and 200 mcg adenosine bolus in the FFR procedure of RCA caused two findings in our research. First, in the 262 lesions that underwent the FFR procedure of RCA, we discovered that the lowest Pd/Pa occurred at the higher dose of adenosine (150 and 200 mcg), about two-thirds of these lesions, whereas in the 793 lesions that underwent the FFR procedure of LCA, the lowest Pd/Pa occurred at the higher dose of adenosine in only half of them (Supplementary Figure S3a). Second, as the clinicians decided on further treatment between continuing medication or considering further revascularization, the discordant ischemic results of Pd/Pa between the higher-dose and the lower-dose adenosine were much more critical than the dose in which the lowest Pd/Pa occurred. In this regard, our findings revealed that discordant ischemic Pd/Pa results were more common in the FFR procedure of RCA than in the FFR procedure of LCA (5.7% vs. 4.0%, respectively) (Supplementary Figure S3b).

The present research differed from previous studies with direct intracoronary adenosine FFR measurement. In the past, the dose of direct intracoronary adenosine was very low, as in the study of Wilson, R. et al. [10] in 1990, with the dose of 16 mcg in LCA and 12 mcg in RCA. As time passed, the dosing of intracoronary adenosine was higher as 150 mcg in both LCA and RCA by Rioufol G. et al. [14] in 2005. The dose was as high as 720 mcg in both LCA and RCA in the study of De Luca G. et al. [15]. In 2015, Adjedj J. et al. [16] measured coronary doppler flow velocity, instead of FFR, after using nine doses of intracoronary adenosine bolus (4, 12, 20, 60, 100, 160, 200, 300, and 500 mcg) in 20 LCA and 10 RCA with less than 20% stenosis or near-normal coronary artery and found that intracoronary adenosine doses of 200 mcg for LCA and 100 mcg for RCA were adequate to achieve more than 95% of maximal coronary hyperemia. Finally, the ACC referenced the recommended dose of 200 mcg for LCA and 100 mcg for RCA for direct intracoronary FFR measurement in 2017 [20]. After the 2017 ACC recommendation, there still have been studies with adenosine higher dose than the recommendation, for example, in the study of Jong CB. et al. [19], with a dose of 800 mcg in both the left and right coronary arteries.

This research might be helpful in FFR practice in the following ways. Firstly, in applying the current predictive model as a screening tool before the reference test of the FFR procedure. In this research, we found that the negative predictive value by using our final four predictors as a predictive model in ischemic coronary lesions was 86.1%, and the negative predictive value using the current predictive model in all coronary lesions that had negative results after lower-dose adenosine bolus would be 96.7%. Secondly, giving an adequate dose of adenosine boluses during the FFR procedure of coronary stenotic lesions could reduce underestimation of the magnitude of coronary ischemia and improve the accuracy of the decision for further revascularization. From our observational data, the most potent predictor in the current model was the FFR procedure of RCA. In addition, giving only a 50 or 100 mcg adenosine bolus during FFR measurement, especially in a young male with low atherosclerotic risk factors, i.e., non-smoking status, could miss the actual ischemic results in 5.7% of the RCA lesions (Supplementary Figure S3b).

The main limitation of this research includes the following. First, due to the nature of the retrospective analysis, the numbers of each coronary artery in which FFR measurement was performed were unequal. Second, even though our direct intracoronary adenosine injection protocol was the four increasing doses, the number of coronary stenotic lesions that underwent the complete four doses was still lower than the incomplete four doses protocol. However, we performed a sensitivity analysis in a subgroup of coronary lesions that received only the complete four increasing adenosine boluses, and the results were concordant with the main results (Supplementary Tables S2-1℃S2-3). Third, this was a single-centered study; our final predictors require external validation as prospective research to confirm the results.

## 5. Conclusions

From our results, factors predicting 150 and 200 mcg adenosine requirement in demonstrating myocardial ischemia from coronary stenotic lesions during four increasing intracoronary adenosine bolus FFR measurements were males with a younger age, non-smoking status, and an FFR procedure of RCA. 

## Figures and Tables

**Table 1:** Site of coronary stenotic lesions that underwent FFR measurement.

Site of Coronary Lesions FFR Performed		n (n%)
LMCA		19 (1.8)
LAD	total	625 (59.2)
	proximal	299 (28.3)
	mid	314 (29.8)
	distal	12 (1.1)
LCX	total	149 (14.1)
	proximal	81 (7.7)
	mid	62 (5.9)
	distal	6 (0.6)
RCA	total	262 (24.8)
	proximal	92 (8.7)
	mid	142 (13.5)
	distal	28 (2.7)

LMCA—left main coronary artery; LAD—left anterior descending artery; LCX—left circumflex artery; RCA—right coronary artery.

**Table 2:** Percent diameter stenosis of coronary stenotic lesions underwent FFR measurement.

Diameter Stenosis	n (n%)
30–40%	71 (6.73)
41–50%	205 (19.43)
51–60%	318 (30.14)
61–70%	380 (36.02)
71–80%	80 (7.58)
81–90%	1 (0.09)

**Table 3:** Comparison of clinical and angiographic characteristics between coronary stenotic lesions with FFR ≤ 0.8 at adenosine 150, 200 mcg and lesions with FFR ≤ 0.8 at adenosine 50, 100 mcg.

Characteristics	FFR ≤ 0.8 at Adenosine 150, 200 mcg (n = 47 Lesions)	FFR ≤ 0.8 at Adenosine 50, 100 mcg (n = 186 Lesions)	*p-*Value
Clinical, n (n%)			
Age (years, mean ± SD)	60.8 ± 10.6	63.0 ± 10.3	0.194
Age group			0.012
Age < 65	36 (76.6)	104 (55.9)	
Age ≥ 65	11 (23.4)	82 (44.1)	
Male	38 (80.9)	125 (67.2)	0.076
BMI (kg/m^2^, mean ± SD)	25.5 ± 4.4	25.1 ± 3.7	0.599
BMI group			0.414
BMI < 25	20 (42.6)	94 (50.5)	
BMI ≥ 25	27 (57.5)	92 (49.5)	
DM	16 (34.0)	75 (40.3.7)	0.505
HT	38 (80.8)	165 (88.7)	0.151
HLP	44 (93.6)	185 (99.5)	0.027
Smoking	22 (46.8)	97 (52.1)	0.519
Presentation			0.775
CCS	42 (89.4)	170 (91.4)	
UA/NSTEMI within 3 months	5 (10.6)	16 (8.6)	
Angiographic, n (n%)			
Baseline Pd/Pa (mean ± SD)	0.93 ± 0.02	0.91 ± 0.05	0.095
FFR (mean ± SD)	0.78 ± 0.04	0.75 ± 0.05	<0.001
Left vs. Right FFR			0.004
FFR of LMCA/LAD/LCX	32 (68.1)	162 (87.1)	
FFR of RCA	15 (31.9)	24 (12.9)	
Part of vessels			1.000
Proximal lesion	20 (42.6)	79 (42.5)	
Mid/Distal lesion	27 (57.4)	107 (57.5)	
Percent stenosis (mean ± SD)	66.6 ± 11.1	69.1 ± 9.2	0.114
Percent stenosis group			0.257
<70% in non-LM, <50% in LM	15 (31.9)	43 (23.1)	
≥70% in non-LM, ≥50% in LM	32 (68.1)	143 (76.9)	
TVD	22 (46.8)	76 (40.9)	0.510

Pd/Pa—ratio of pressure distal to coronary lesion divided by pressure of aorta; BMI—body mass index; DM—diabetes mellitus; HT—hypertension; HLP—hyperlipidemia; CCS—chronic coronary syndrome; UA—unstable angina; NSTEMI—non-ST elevation myocardial infarction; FFR—fractional flow reserve; LMCA, LM—left main coronary artery; LAD—left anterior descending artery; LCX—left circumflex artery; RCA—right coronary artery; TVD—triple vessels coronary artery disease.

**Table 4:** Crude and adjusted odds ratio of predictors in which the FFR ≤ 0.8 occurred at adenosine 150, 200 mcg.

Variables	Crude		Adjusted	
	OR (95% CI)	*p*-Value	OR (95% CI)	*p*-Value
Age < 65 yrs	2.58 (1.24–5.39)	0.012	2.26 (0.98–5.22)	0.056
Male	2.06 (0.94–4.54)	0.073	3.18 (1.08–9.37)	0.036
BMI ≥ 25 kg/m^2^	1.38 (0.72–2.63)	0.330	1.77 (0.80–3.94)	0.161
Non-DM	1.31 (0.67–2.56)	0.432	1.48 (0.69–3.16)	0.315
Non-HT	1.86 (0.79–4.39)	0.156	1.50 (0.57–3.96)	0.413
Non-HLP	12.61 (1.27–124.79)	0.030	23.85 (3.70–153.88)	0.001
Non-smoking	1.24 (0.65–2.35)	0.514	3.02 (1.20–7.62)	0.019
UA/NSTEMI within 3 months	1.26 (0.44–3.66)	0.664	1.69 (0.48–5.93)	0.411
FFR of RCA	3.16 (1.49–6.70)	0.003	4.35 (1.72–11.03)	0.002
Mid/Distal lesion	1.00 (0.52–1.91)	0.992	0.70 (0.33–1.46)	0.340
Non-sig. CAG	1.56 (0.77–3.15)	0.216	1.65 (0.70–3.87)	0.253
TVD	1.27 (0.67–2.43)	0.462	1.48 (0.71–3.10)	0.300

BMI—body mass index; DM—diabetes mellitus; HT—hypertension; HLP—hyperlipidemia; UA—unstable angina; NSTEMI—non-ST elevation myocardial infarction; FFR—fractional flow reserve; RCA—right coronary artery; Non-sig. CAG—non-significant stenotic lesion from coronary angiogram evaluated by visual estimation; TVD—triple vessels coronary artery disease.

**Table 5:** Final four predictors in parsimonious model, adjusted odds ratio, and AuROC.

Predictors	Adjusted OR (95% CI)	*p*-Value	AuROC (95% CI)
Age < 65 yrs	2.51 (1.13–5.58)	0.024	0.60 (0.57–0.64)
Male	3.04 (1.15–8.06)	0.025	0.57 (0.54–0.60)
Non-smoking	2.42 (1.05–5.61)	0.039	0.53 (0.49–0.57)
FFR of RCA	3.31 (1.44–7.62)	0.005	0.60 (0.56–0.63)

FFR—fractional flow reserve; RCA—right coronary artery.

## Data Availability

The data in this study are available on request from the corresponding author.

## Supplementary Materials

The following supporting information can be downloaded at: https://www.mdpi.com/article/10.3390/diagnostics12092076/s1, Figure S1: Baseline Pd/Pa, Pd/Pa50, Pd/Pa100, Pd/Pa150, and Pd/Pa200 in FFR measurement of right coronary artery (262 lesions) and left coronary artery (793 lesions). RCA: right coronary artery; LCA: left coronary artery. Figure S2: Cumulative frequency of ischemic FFR (FFR ≤0.8) in FFR of left and right coronary artery. FFR: fractional flow reserve; mcg: microgram. Figure S3: Comparison of the FFR of RCA and the FFR of LM/LAD/LCX. The number of coronary stenotic lesions in which (a) the lowest Pd/Pa value obtained during the lower (50, 100 mcg) and the higher (150, 200 mcg) adenosine bolus dose.; (b) the FFR ≤0.8 occurred at the higher (150, 200 mcg) adenosine bolus. FFR: fractional flow reserve; RCA: right coronary artery; Pd/Pa: ratio of pressure distal to coronary lesion divided by pressure of aorta; mcg: microgram; LM: left main coronary artery; LAD: left anterior descending artery; LCX: left circumflex artery. Table S1: Sample size estimation from 228 coronary stenotic lesions in pilot survey data. Table S2-1: Sensitivity analysis in 391 coronary stenotic lesions underwent the complete four doses intracoronary adenosine bolus FFR measurement: comparison of clinical and angiographic characteristics between lesions with FFR ≤0.8 at adenosine 150, 200 mcg and lesions with FFR ≤0.8 at adenosine 50, 100 mcg. Table S2-2: Sensitivity analysis in 391 coronary stenotic lesions underwent the complete four doses intracoronary adenosine bolus FFR measurement: crude and adjusted odds ratio of predictors in which the FFR ≤0.8 occurred at adenosine 150, 200 mcg. Table S2-3: Sensitivity analysis in 391 coronary stenotic lesions underwent the complete four doses intracoronary adenosine bolus FFR measurement: final four predictors in parsimonious model, adjusted odds ratio, and AuROC.
