# Supplementary material for: Factors Predicting 150 and 200 Microgram Adenosine Requirement during Four Increasing Doses of Intracoronary Adenosine Bolus Fractional Flow Reserve Assessment
Source: Diagnostics (Basel). 2022 Aug 27;12(9):2076. doi: 10.3390/diagnostics12092076 (PMC9498048; doi:10.3390/diagnostics12092076)
Supplement: Supplementary file 1 [file diagnostics-12-02076-s001.zip › diagnostics-1827297-supplementary.pdf]

Supplementary material

Supplementary Table S1. Sample size estimation from 228 coronary stenotic lesions in pilot survey data.

Characteristics	FFR obtained at 150, 200 mcg adenosine (n=117 lesions)	FFR obtained at 50, 100 mcg adenosine (n=111 lesions)	n requirement
**Clinical** (n%)			
Age (years, mean±SD)	64.05±9.64	61.89±9.44	307/307
Age<65	55.56	61.26	1,208/1,208
Age≥65	44.44	38.74	1,208/1,208
Male	70.09	60.36	396/396
BMI (kg/m^2, mean±SD)	25.42±4.30	24.97±4.00	1,337/1,337
BMI<25	50.43	60.36	413/413
BMI≥25	49.57	39.64	413/413
DM	31.62	41.44	397/397
CCS	93.16	90.99	2,523/2,523
Unstable angina	2.56	4.50	1,521/1,521
NSTEMI	4.27	4.5	125,284/125,284
**Angiographic** (n%)			
LMCA	4.27	4.50	125,284/125,284
LAD	58.97	61.26	7,264/7,264
LCX	6.84	12.61	447/447
LM+LAD+LCX	70.08	78.37	460/460
RCA	29.91	21.62	460/460
Proximal part	47.32	45.28	9,476/9,476
Mid part	47.32	50	5,534/5,534
Distal part	5.36	4.72	18,653/18,653

mcg: microgram; BMI: body mass index; DM: diabetes mellitus; CCS: chronic coronary syndrome; NSTEMI: non-ST elevation myocardial infarction; LMCA: left main coronary artery; LAD: left anterior descending artery; LCX: left circumflex artery; RCA: right coronary artery

Supplementary Table S2-1. Sensitivity analysis in 391 coronary stenotic lesions underwent the complete four doses intracoronary adenosine bolus FFR measurement: comparison of clinical and angiographic characteristics between lesions with FFR ≤0.8 at adenosine 150, 200 mcg and lesions with FFR ≤0.8 at adenosine 50, 100 mcg.

**Characteristics**	**FFR ≤0.8 at adenosine 150, 200 mcg (n=20 lesions)**	**FFR ≤0.8 at adenosine 50, 100 mcg (n=53 lesions)**	***p* Value**
**Clinical,** n (n%)			
Age (years, mean±SD)	60.4±10.8	62.9±9.2	0.322
Age group			0.102
Age <65	16 (80.0)	30 (56.6)	
Age ≥65	4 (20.0)	23 (43.4)	
Male	17 (85.0)	32 (60.4)	0.054
BMI (kg/m^2, mean±SD)	24.4±3.3	25.3±3.6	0.368
BMI group			0.607
BMI <25	9 (45.0)	28 (52.8)	
BMI ≥25	11 (55.0)	25 (47.2)	
Non-DM	12 (60.0)	30 (56.6)	1.000
Non-HT	5 (25.0)	6 (11.3)	0.160
Non-HLP	2 (10.0)	0 (0)	0.072
Nonsmoker	11 (55.0)	26 (49.1)	0.794
Presentation			0.384
CCS	17 (85.0)	49 (92.4)	
UA/NSTEMI	3 (15.0)	4 (7.6)	
**Angiographic,** n (n%)			
Baseline Pd/Pa (mean±SD)	0.93±0.02	0.92±0.02	0.157
FFR (mean±SD)	0.78±0.03	0.75±0.04	0.015
Left vs. Right FFR			<0.001
FFR of LMCA/LAD/LCX	11 (55.0)	50 (94.3)	
FFR of RCA	9 (45.0)	3 (5.7)	
Part of vessels			1.000
Proximal lesion	9 (45.0)	25 (47.2)	
Mid/Distal lesion	11 (55.0)	28 (52.8)	
Percent stenosis (mean±SD)	66.5±11.8	68.3±9.1	0.492
Percent stenosis group			0.766
<70% non-LM, <50% in LM	6 (30)	13 (24.5)	
≥70% non-LM, ≥50% in LM	14 (70)	40 (75.5)	
TVD	8 (40.0)	21 (39.6)	1.000

Pd/Pa: ratio of pressure distal to coronary lesion divided by pressure of aorta; BMI: body mass index; DM: diabetes mellitus; HT: hypertension; HLP: hyperlipidemia; CCS: chronic coronary syndrome; UA: unstable angina; NSTEMI: non-ST elevation myocardial infarction; FFR: fractional flow reserve; LMCA, LM: left main coronary artery; LAD: left anterior descending artery; LCX: left circumflex artery; RCA: right coronary artery; TVD: triple vessels coronary artery disease

Supplementary Table S2-2. Sensitivity analysis in 391 coronary stenotic lesions underwent the complete four doses intracoronary adenosine bolus FFR measurement: crude and adjusted odds ratio of predictors in which the FFR ≤0.8 occurred at adenosine 150, 200 mcg.

**Variables**	**Crude**		**Adjusted**	
	**OR (95% CI)**	***p* Value**	**OR (95% CI)**	***p* Value**
Age <65 yrs	3.07 (0.90-10.51)	0.074	8.34 (0.68-101.75)	0.096
Male	3.72 (0.96-14.41)	0.057	85.14 (8.36-867.35)	<0.001
BMI ≥25 kg/m^2	1.37 (0.48-3.87)	0.554	3.37 (0.42-27.23)	0.255
Non-DM	1.15 (0.40-3.30)	0.795	3.00 (0.49-18.25)	0.233
Non-HT	2.61 (0.69-9.88)	0.157	2.73 (0.34-21.65)	0.342
Non-HLP	-	-	-	-
Nonsmoker	1.27 (0.45-3.59)	0.653	84.14 (4.79-1478.57)	0.002
UA/NSTEMI	2.16 (0.43-10.78)	0.347	14.79 (1.12-195.13)	0.041
FFR of RCA	13.64 (3.13-59.35)	<0.001	129.10 (5.56-2995.40)	0.002
Mid/Distal lesion	1.09 (0.39-3.09)	0.869	1.02 (0.25-4.26)	0.976
Non-sig. CAG	1.32 (0.42-4.17)	0.638	4.92 (0.57-42.15)	0.146
TVD	1.02 (0.35-2.93)	0.977	1.05 (0.19-5.89)	0.958

Pd/Pa: ratio of pressure distal to coronary lesion divided by pressure of aorta; BMI: body mass index; DM: diabetes mellitus; HT: hypertension; HLP: hyperlipidemia; UA: unstable angina; NSTEMI: non-ST elevation myocardial infarction; FFR: fractional flow reserve; RCA: right coronary artery; Non-sig. CAG: non-significant stenotic lesion from coronary angiogram evaluated by visual estimation; TVD: triple vessels coronary artery disease

Supplementary Table S2-3. Sensitivity analysis in 391 coronary stenotic lesions underwent the complete four doses intracoronary adenosine bolus FFR measurement: final four predictors in parsimonious model, adjusted odds ratio, and AuROC.

Predictors	Adjusted OR (95% CI)	*p* Value	AuROC (95% CI)
Age <65 yrs	5.37 (0.94-30.54)	0.058	0.62 (0.56-0.67)
Male	15.71 (2.65-93.21)	0.002	0.62 (0.57-0.67)
Nonsmoker	8.90 (1.61-49.10)	0.012	0.53 (0.47-0.59)
FFR of RCA	20.29 (3.73-110.43)	<0.001	0.70 (0.64-0.75)

FFR: fractional flow reserve; RCA: right coronary artery

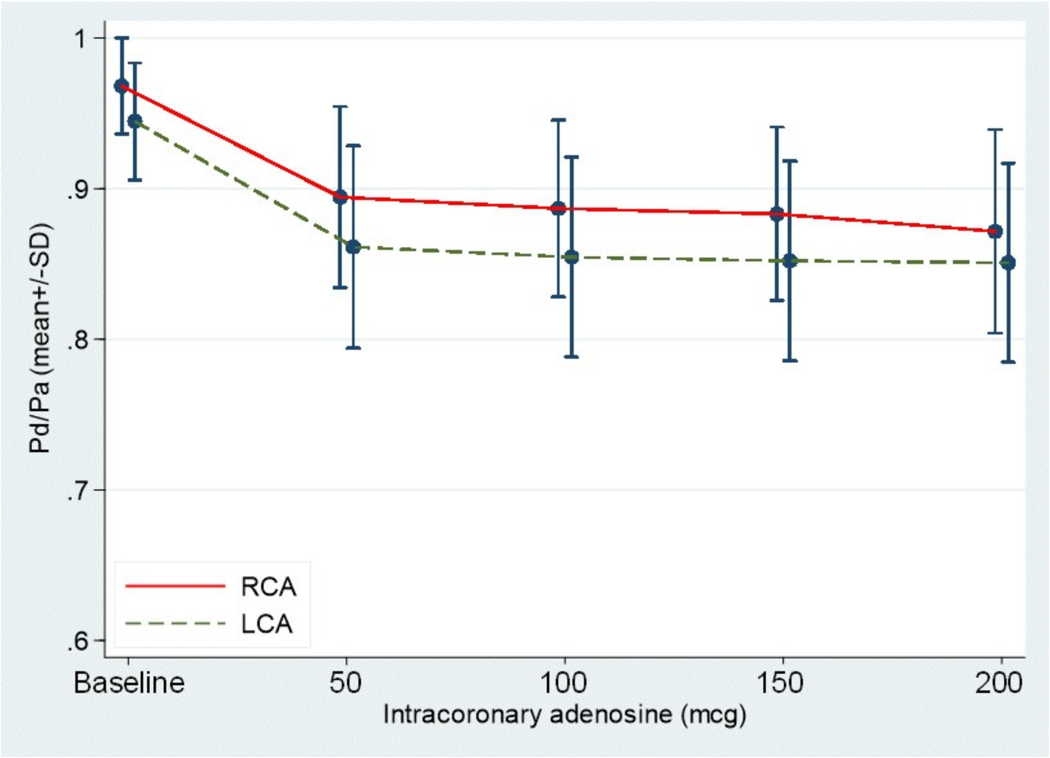

Supplementary Figure S1. Baseline Pd/Pa, Pd/Pa_{50}, Pd/Pa_{100}, Pd/Pa_{150}, and Pd/Pa_{200} in FFR measurement of right coronary artery (262 lesions) and left coronary artery (793 lesions). RCA: right coronary artery; LCA: left coronary artery.

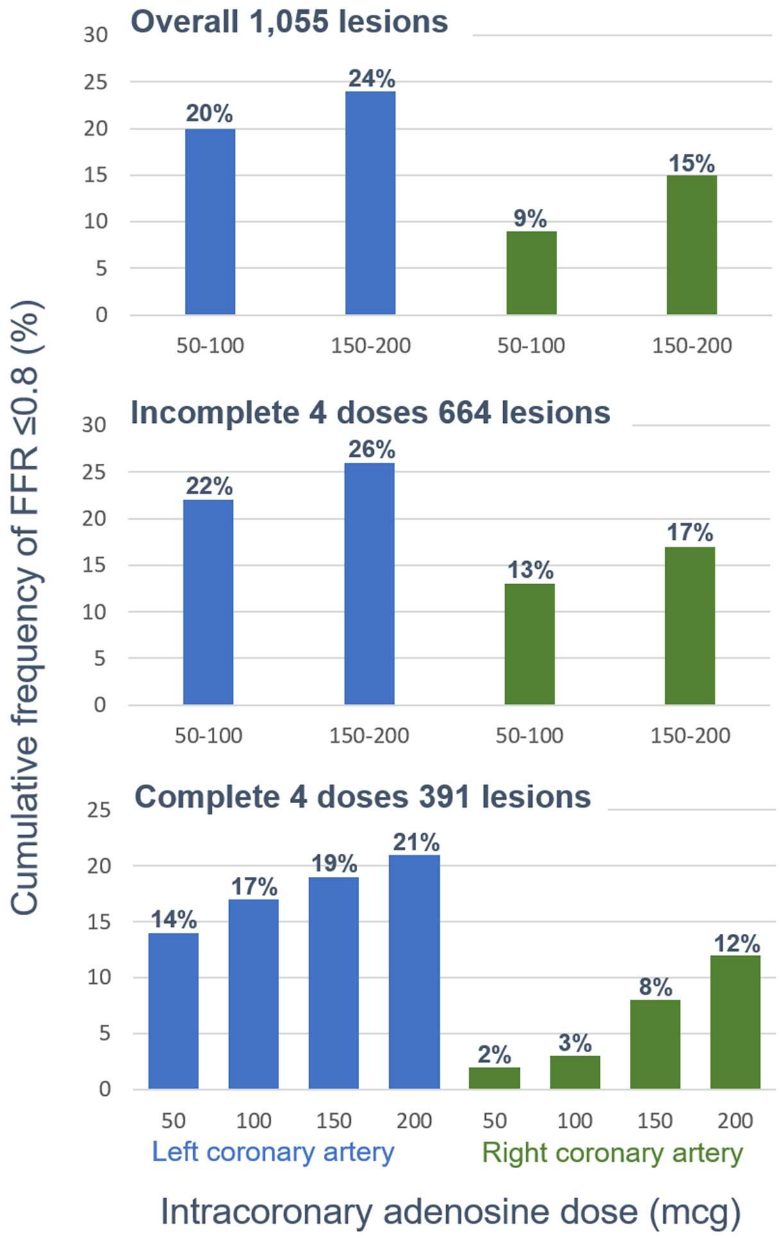

Supplementary Figure S2. Cumulative frequency of ischemic FFR (FFR ≤0.8) in FFR of left and right coronary artery. FFR: fractional flow reserve; mcg: microgram.

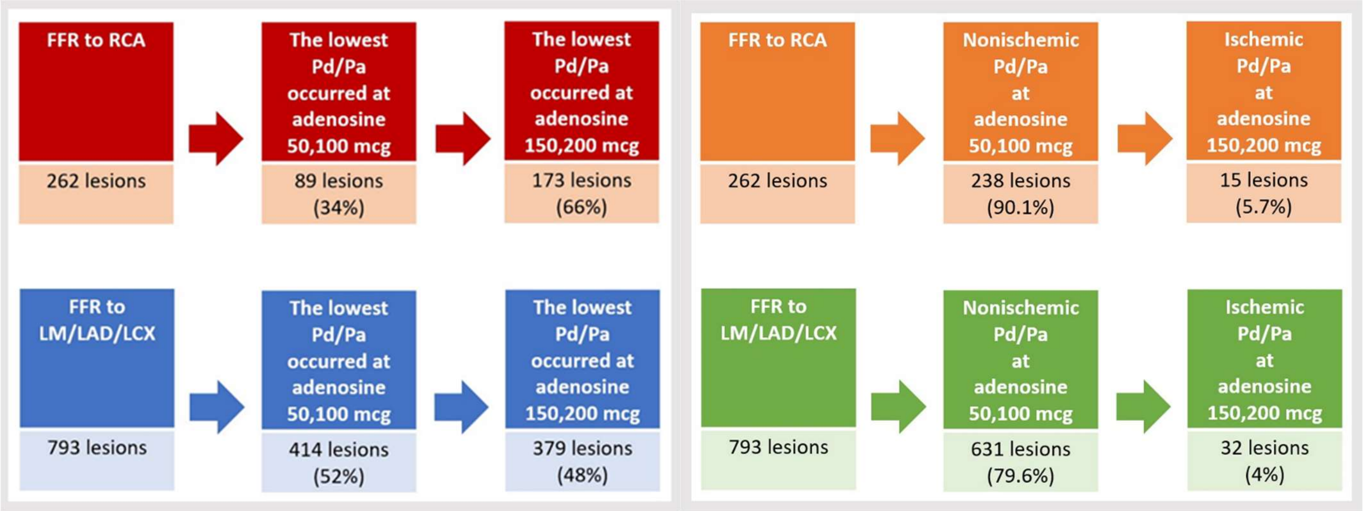

(a) (b)

Supplementary Figure S3. Comparison of the FFR of RCA and the FFR of LM/LAD/LCX. The number of coronary stenotic lesions in which (a) the lowest Pd/Pa value obtained during the lower (50, 100 mcg) and the higher (150, 200 mcg) adenosine bolus dose.; (b) the FFR ≤0.8 occurred at the higher (150, 200 mcg) adenosine bolus. FFR: fractional flow reserve; RCA: right coronary artery; Pd/Pa: ratio of pressure distal to coronary lesion divided by pressure of aorta; mcg: microgram; LM: left main coronary artery; LAD: left anterior descending artery; LCX: left circumflex artery.
